# Response to Letter to the Editor From Behar-Cohen et al.: The Cortisol Response of Male and Female Choroidal Endothelial Cells: Implications for Central Serous Chorioretinopathy

**DOI:** 10.1210/clinem/dgab910

**Published:** 2021-12-18

**Authors:** Joost Brinks, Elon H C van Dijk, Robbert G E Notenboom, Paul H A Quax, Camiel J F Boon, Onno C Meijer

**Affiliations:** 1 Department of Ophthalmology, Leiden University Medical Center, 2300 RC Leiden, the Netherlands; 2 Department of Anatomy and Embryology, Leiden University Medical Center, 2300 RC Leiden, the Netherlands; 3 Department of Vascular Surgery, Leiden University Medical Center, 2300 RC Leiden, the Netherlands; 4 Einthoven Laboratory for Experimental Vascular Medicine, Leiden University Medical Center, 2300 RC Leiden, the Netherlands; 5 Department of Ophthalmology, Amsterdam University Medical Centers, University of Amsterdam, 1100 DD Amsterdam-Zuidoost, the Netherlands; 6 Department of Medicine, Division of Endocrinology and Metabolism, Leiden University Medical Center, 2300 RC Leiden, the Netherlands

Dear Editor,

In their letter (1), Behar-Cohen and colleagues raise issues about our work on choroidal endothelial cells in relation to corticosteroid effects and central serous chorioretinopathy (CSC), an eye disease in which fluid accumulates underneath the retina at sites of choroidal vascular dysfunction and hyperpermeability, leading to impaired vision (2).

Corticosteroid hormones constitute a major risk factor for the development of CSC. Corticosteroids have 2 known receptor types: mineralocorticoid and glucocorticoid receptors (MR/GR). The MR is of interest, for example, because patients with hyperaldosteronism can present with CSC symptomatology (3). However, MR antagonists have been proven to be ineffective as a treatment for CSC in a large prospective, randomized, double-blind, placebo-controlled trial (4). Given the leaking endothelial barrier in CSC, we studied MR/GR presence in the human choroidal vasculature, using immunofluorescence. We detected GR but not MR immunoreactivity in choroidal endothelial cells. Our colleagues claim that these results are likely false negative for MR, assuming degradation of the protein.

We could not detect choroidal MR immunoreactivity in 6 eyes, collected within 24 hours postmortem—including delays of 3 to 4 hours and 6 hours. In their letter, our colleagues incorrectly rephrased this to “around 24 hours delay”. Nevertheless, MR may have degraded in our samples. Hence, we included positive control stainings. We have reliably detected MR immunoreactivity in the expected places in a diversity of human tissue sections including human kidney and (not shown) hippocampus. Importantly, the human eye offers a positive control *within* tissue sections: in the retina we did stain the MR protein. It is however true that we cannot exclude degradation specifically in endothelial cells, or epitope masking in these cells. We did observe essentially identical cellular presence of immunoreactivity with the 1D5 MR antibody, kindly provided by Dr. Gomez-Sanchez (5).

Behar-Cohen et al seem to find a crucial argument in discrepancies between our results and published literature. However, to the best of our knowledge, all previous work on choroidal MR presence was done in rodents. To demand that a human study yields identical results to work in rats seems to us an untenable position. In fact, there are well-known anatomical differences between rodent and human eyes, for example, the absence/presence of a macula. Even with its intrinsic limitations, the use of human tissue to understand human disease seems to us a particular strength of our study.

Our colleagues then argue that our experiments are anyhow futile in relation to understanding CSC. Of course, and as mentioned (2), CSC may well result from steroid effects in the many cell types, including other tissues than the choroid. Yet, given that choroidal vascular hyperpermeability is a key aspect of CSC, we consider it valid and obvious to hypothesize that corticosteroids effects on choroidal endothelial cells play a role. Of note, Behar-Cohen and colleagues endorsed this idea themselves (6). In fact, our data suggest plenty of links to nitric oxide production and vascular permeability (2). Whether these responses are truly involved in CSC will have to be established with further research.

## Abbreviations

CSC: central serous chorioretinopathy
GR: glucocorticoid receptor
MR: mineralocorticoid receptor

## References

[1] Behar-Cohen F , Zhao M, Jaisser F. Letter to the editor from Behar-Cohen, et al: “The Cortisol Response of Male and Female Choroidal Endothelial Cells: Implications for Central Serous Chorioretinopathy”. 10.1210/clinem/dgab908 34922389

[2] Brinks J , van Dijk EHC, Kiełbasa SM, et al. The cortisol response of male and female choroidal endothelial cells: implications for central serous chorioretinopathy. J Clin Endocrinol Metab. 2021. doi:10.1210/clinem/dgab670 PMC8764349 34546342

[3] van Dijk EH , Nijhoff MF, de Jong EK, Meijer OC, de Vries AP, Boon CJ. Central serous chorioretinopathy in primary hyperaldosteronism. Graefes Arch Clin Exp Ophthalmol. 2016;254(10):2033-2042. 27393297 10.1007/s00417-016-3417-8 PMC5045484

[4] Lotery A , Sivaprasad S, O’Connell A, et al.; VICI trial investigators. Eplerenone for chronic central serous chorioretinopathy in patients with active, previously untreated disease for more than 4 months (VICI): a randomised, double-blind, placebo-controlled trial. Lancet. 2020;395(10220):294-303. 31982075 10.1016/S0140-6736(19)32981-2

[5] Gomez-Sanchez CE , Warden M, Gomez-Sanchez MT, Hou X, Gomez-Sanchez EP. Diverse immunostaining patterns of mineralocorticoid receptor monoclonal antibodies. Steroids. 2011;76(14):1541-1545. 21945398 10.1016/j.steroids.2011.09.004 PMC3217130

[6] Daruich A , Matet A, Dirani A, et al. Central serous chorioretinopathy: recent findings and new physiopathology hypothesis. Prog Retin Eye Res. 2015;48:82-118. 26026923 10.1016/j.preteyeres.2015.05.003

